# miR-100-3p inhibits the adipogenic differentiation of hMSCs by targeting PIK3R1 via the PI3K/AKT signaling pathway

**DOI:** 10.18632/aging.104074

**Published:** 2020-11-20

**Authors:** Tao Wang, Donghuo Zhong, Zhongjun Qin, Shan He, Ying Gong, Weidong Li, Xingnuan Li

**Affiliations:** 1 Key Laboratory of System Bio-Medicine of Jiangxi Province, Jiujiang University, Jiujiang 332000, China

**Keywords:** microRNA-100-3p, hMSCs, adipogenic differentiation, PIK3R1, PI3K/AKT signaling pathway

## Abstract

MicroRNAs play an important role in the adipogenic differentiation of human bone marrow mesenchymal stem cells (hMSCs). How miR-100-3p influences such adipogenesis, however, remains uncertain. In this study, hMSC adipogenic differentiation was associated with miR-100-3p downregulation, and overexpressing this miRNA inhibited adipogenesis and the expression of adipogenic marker genes. Through bioinformatics approaches, miR-100-3p can bind the 3'-untranslated region (3′-UTR) of the mRNA encoding phosphoinositide 3-kinase regulatory subunit 1 (PIK3R1) such that miR-100-3p overexpression resulted in significant reductions in PIK3R1 expression. Importantly, overexpressing PIK3R1 was sufficient to reverse the anti-adipogenic effects of miR-100-3p overexpression. PIK3R1 is a critical component of the PI3K/AKT signaling pathway, and miR-100-3p overexpression resulted in reduced AKT phosphorylation in the context of adipogenesis. In addition, the adipogenic differentiation of hMSCs in which miR-100-3p was overexpressed was further enhanced upon treatment with the PI3K/AKT agonist 740Y-P relative to miR-100-3p overexpression alone. Taken together, these findings provide evidence that miR-100-3p inhibits the adipogenic differentiation of hMSCs by targeting PIK3R1 via the PI3K/AKT signaling pathway.

## INTRODUCTION

Adipogenesis is a process whereby preadipocyte precursor cells differentiate into mature adipocytes, which serve as the primary cells responsible for fat storage [1, 2]. Dysregulated adipogenesis can result in obesity and is associated with serious comorbidities including cardiovascular disease and type II diabetes mellitus (T2DM), particularly in elderly populations [3, 4]. Further research into the molecular basis for adipogenesis may thus be a viable approach to preventing or treating obesity and related conditions.

Human bone marrow-derived mesenchymal stem cells (hMSCs) are multipotent stem cells that undergo self-renewal and differentiate into osteocytes, chondrocytes, and adipocytes [5], with the *in vitro* directed adipogenic differentiation of these cells being possible [6]. Such *in vitro*-expanded hMSCs thus represent a valuable model for the study of adipogenesis.

MicroRNAs (miRNAs) are RNAs that lack coding potential, yet are able to bind to complementary 3’-untranslated region (3’-UTR) sequences in target mRNAs and to thereby modulate their post-transcriptional expression [7–9]. Different miRNAs play myriad roles as regulators of metabolic activity, cellular proliferation, and apoptosis [10–12]. There is evidence that miR-100-3p can modulate apoptosis and cell growth in certain cancers [13, 14], but its role in the context of hMSC adipogenic differentiation remains uncertain.

Adipogenesis is a complex multi-stage process that necessitates the coordinated activation of multiple signaling pathways, with phosphatidylinositol 3-kinase (PI3K)/AKT signaling being essential to this differentiation process [6, 15]. PI3K is a heterodimeric protein composed of a p110 subunit and a p85 subunit (also known as PI3K regulatory subunit 1 [PIK3R1]), both of which are vital for normal PI3K signaling activity [16–17].

Herein, we explored the functional importance of miR-100-3p as a regulator of adipogenesis in hMSCs. Through this study, we ultimately determined that this miRNA targets PIK3R1 via the PI3K/AKT signaling pathway, ultimately inhibiting adipogenic differentiation.

## RESULTS

### Evaluation of miR-100-3p expression over the course of hMSC adipogenesis

**Figure 1:**
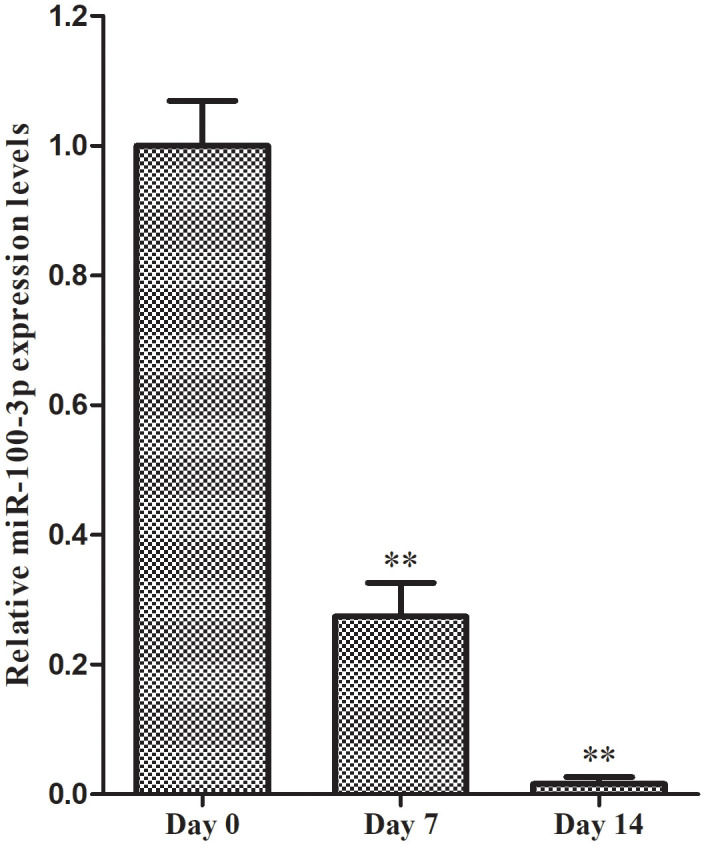
**qRT-PCR was used to monitor miR-100-3p expression during adipogenesis. Data are means ± SD (X±SD, n=3).** **P<0.01 vs. day 0.

We began by culturing hMSCs in adipogenic medium for 7-14 days and assessing miR-100-3p expression over the course of this differentiation period. We found that miR-100-3p expression levels dropped markedly during differentiation by 72.6% and 98.4% on days 7 and 14, respectively, relative to baseline (Figure 1).

### Preparation of hMSCs stably overexpressing miR-100-3p

**Figure 2:**
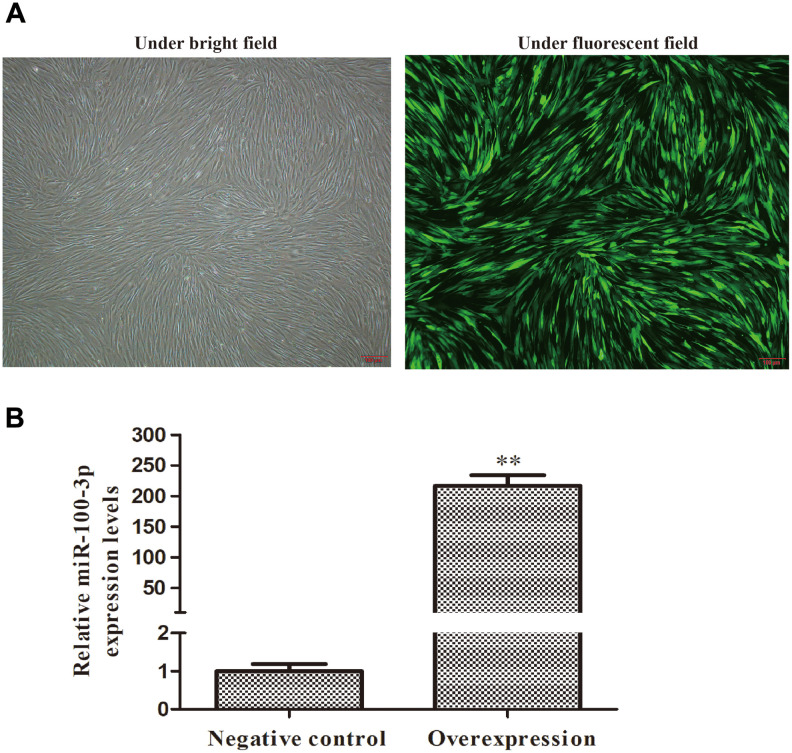
**Screening of stably transduced hMSCs.** Control or miR-100-3p overexpression lentiviral vectors were used to infect hMSCs. (**A**) Lentivirally transduced cells were assessed via light and fluorescent microscopy (4×); scale bar, 100 μm, with a representative image being shown. (**B**) qRT-PCR was used to measure miR-100-3p overexpression. Data are means ± SD (X ± SD, n=3). **P<0.01 vs negative control.

We next transduced hMSCs with lentiviral vectors encoding miR-100-3p or appropriate control constructs prior to adipogenic differentiation. GFP expression was clearly detectable in transduced cells, consistent with stable transduction (Figure 2A). We then used qRT-PCR to assess these cells and confirmed that miR-100-3p was upregulated over 200-fold therein (Figure 2B).

### Overexpression of miR-100-3p suppresses hMSC adipogenesis

**Figure 3:**
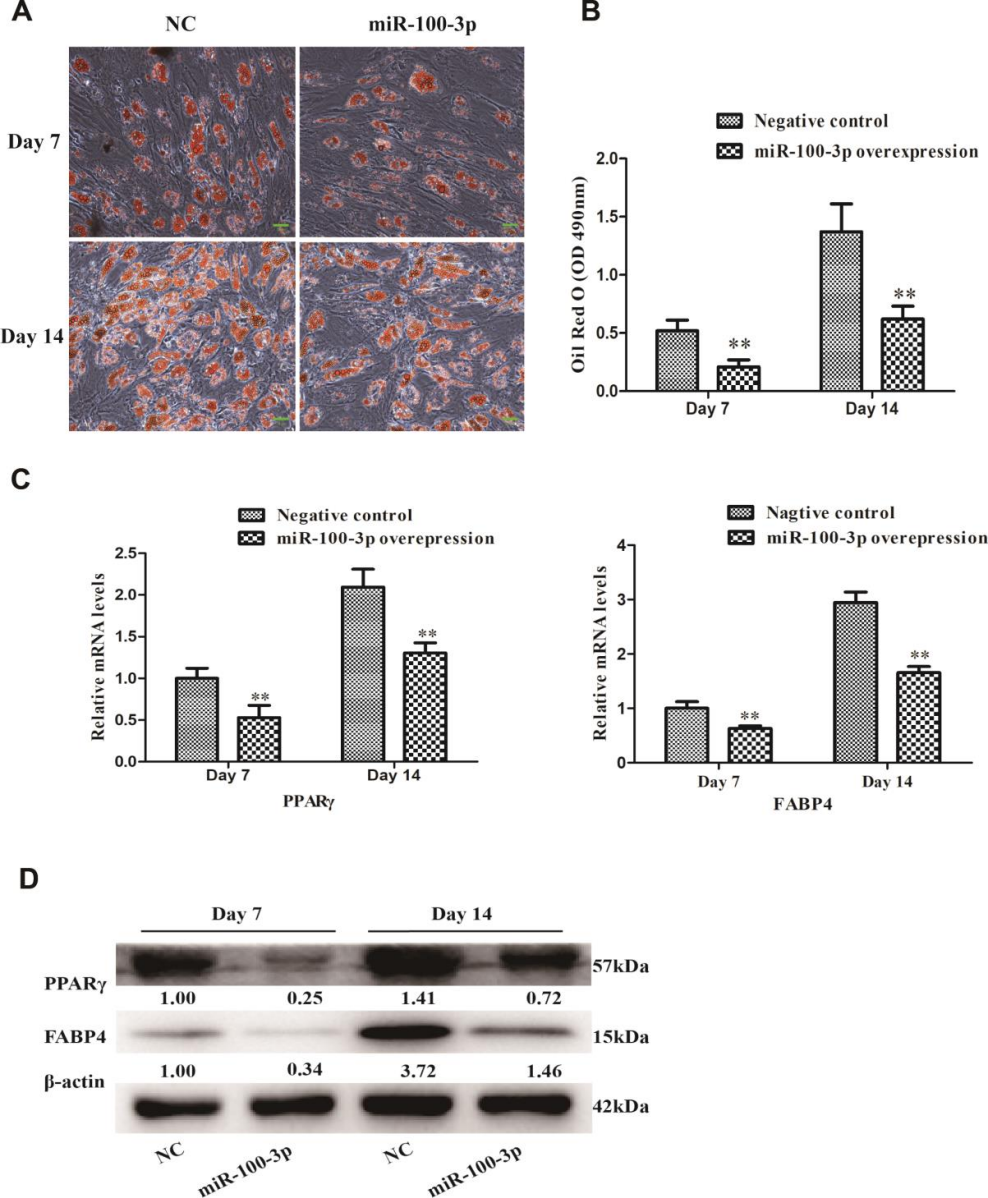
**miR-100-3p overexpression impairs hMSC adipogenesis.** (**A**) Oil Red O staining was used to assess hMSCs over the course of adipogenesis (20×); scale bar, 20μm. (**B**) Quantification of the Oil Red O staining results from these cells. (**C**) qRT-PCR was used to assess PPARγ and FABP4 expression. (**D**) Western blotting was used to assess PPARγ and FABP4 protein levels. Data are means ± SD (*n*=3). **P<0.01 vs. negative controls, respectively. Note: NC: negative control; miR-100-3p: miR-100-3p overexpression

Oil Red O staining was next employed to evaluate the adipogenic differentiation of these transduced cells, revealing that miR-100-3p overexpression markedly inhibited adipogenesis (Figure 3A) and intracellular lipid droplet accumulation (Figure 3B). Consistently, miR-100-3p overexpressing cells expressed lower levels of the adipogenic marker genes PPARγ and FABP4 at the mRNA (Figure 3C) and protein (Figure 3D) levels relative to control cells during adipogenic differentiation.

### miR-100-3p target gene identification

**Figure 4:**
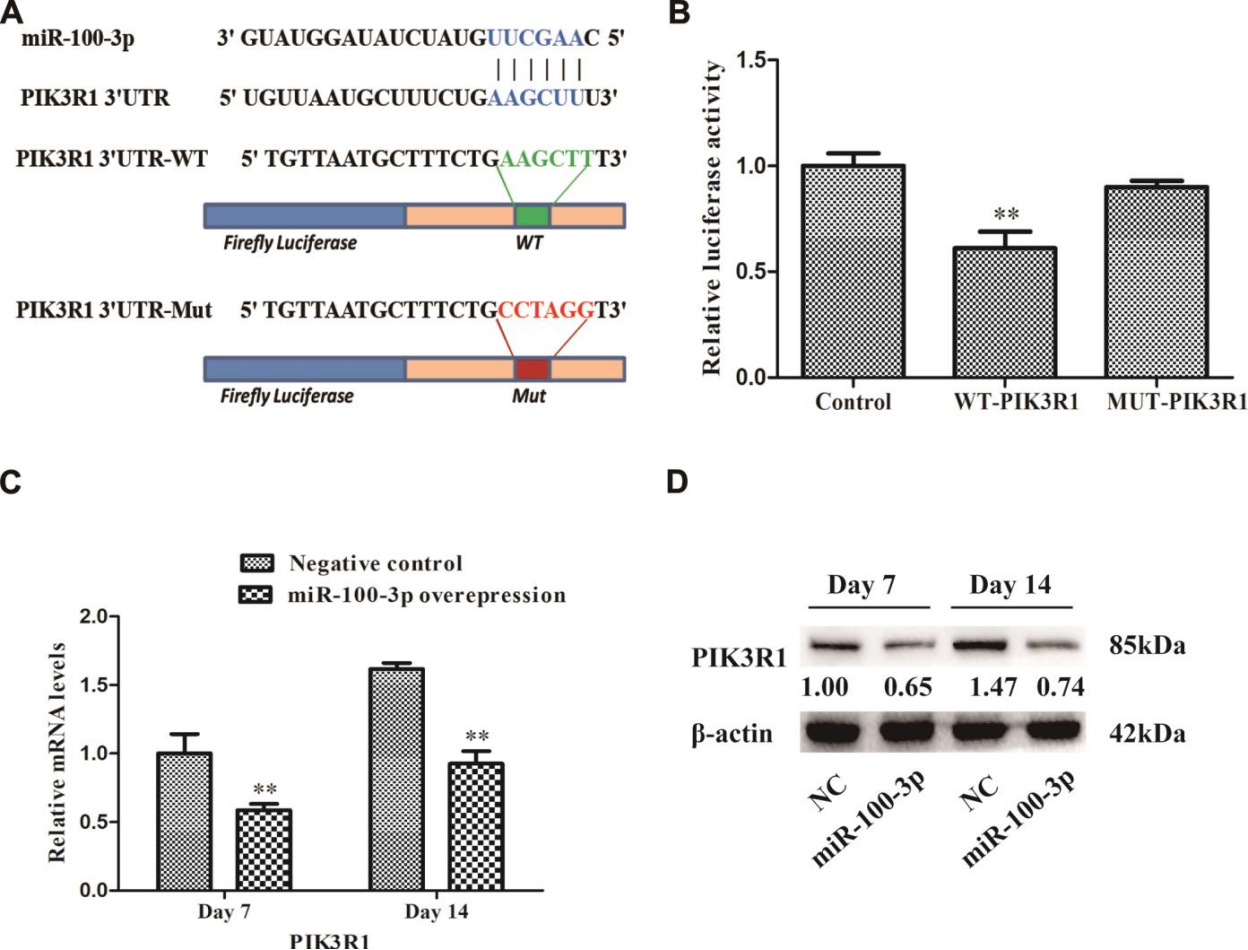
**miR-100-3p specifically binds to the PIK3R1 3′-UTR region.** (**A**) Illustration of the putative site of miR-100-3p binding within the 3’-UTR of PIK3R1, with nucleotides that were mutated for MUT reporter preparation highlighted in red. (**B**) Luciferase activity assay results. (**C**, **D**) The impact of miR-100-3p overexpression on PIK3R1 expression in the context of hMSC adipogenesis was measured via qRT-PCR and Western blotting. Data are means ± SD (*n*=3). **P<0.01 vs. negative controls, respectively. Note: NC: negative control; miR-100-3p: miR-100-3p overexpression.

To identify miR-100-3p target genes, we utilized three predictive algorithms that all identified PIK3R1 as containing a putative 3’-UTR miR-100-3p binding site (Figure 4A). In a luciferase reporter assay, we confirmed that WT PIK3R1 3'-UTR reporter activity was reduced 39% by miR-100-3p mimic co-transfection, whereas no such decrease was observed when a MUT version of this reporter was instead used for this assay (Figure 4B), thus confirming the ability of miR-100-3p to bind to this predicted target sequence.

To further validate this targeting relationship, we measured PIK3R1 expression in hMSCs overexpressing miR-100-3p during adipogenesis (Figure 4C, 4D), revealing a significant decrease in PIK3R1 expression in miR-100-3p-overexpressing cells.

### PIK3R1 overexpression is sufficient to reverse miR-100-3p-mediated suppression of hMSC adipogenesis

**Figure 5:**
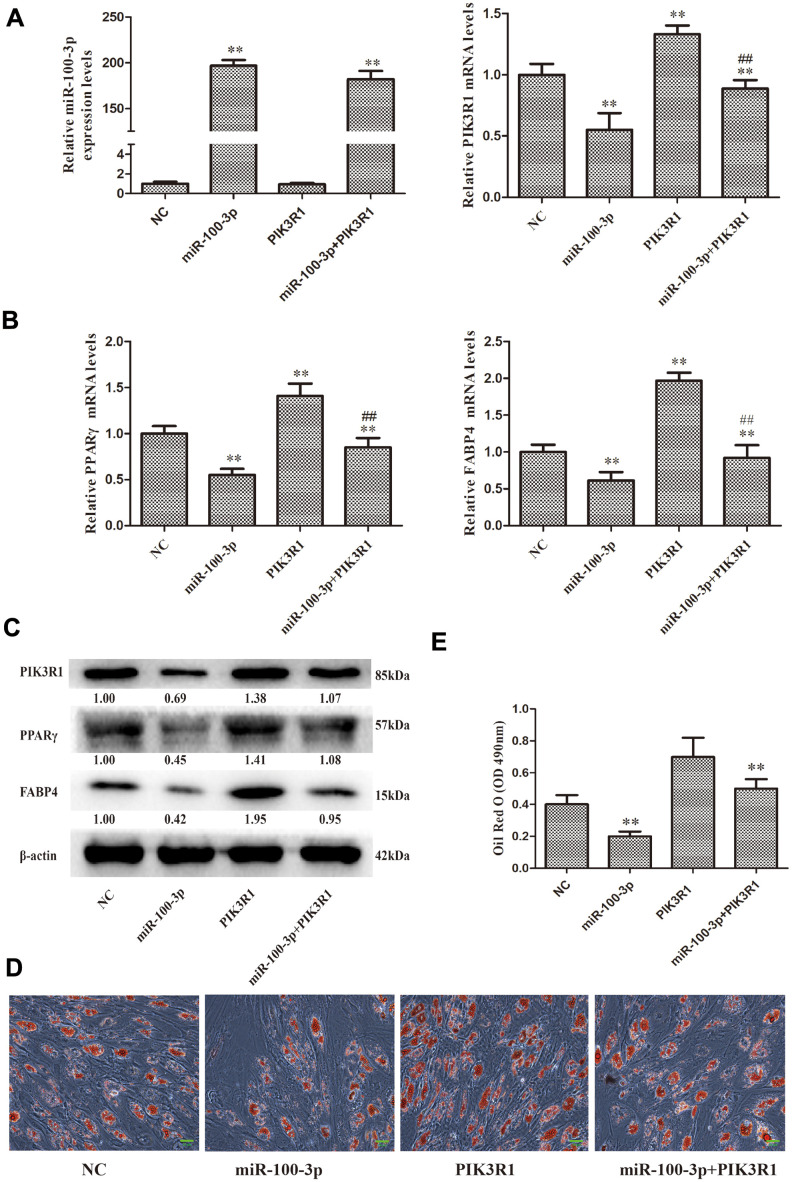
**PIK3R1 overexpression was sufficient to reverse miR-100-3p-mediated suppression of hMSC adipogenesis**. (**A, B**) miR-100-3p, PIK3R, PPARγ, and FABP4 mRNA levels in these cells were assessed. (**C**) Western blotting was used to assess PIK3R1, PPARγ, and FABP4 protein levels. (**D**) Lipid droplets were detected in these cells via Oil Red O staining; scale bar, 20 μm. (**E**) Oil Red O staining intensity differed significantly among study groups. Data were collected on day 7 post-adipogenic induction. Data are means ± SD (X ± SD, n=3). **P<0.01 vs negative control. ^##^*P*<0.01 vs miR-100-3p overexpression. Note: NC: miR-100-3p overexpression negative control and PIK3R1 overexpression negative control; miR-100-3p: miR-100-3p overexpression and PIK3R1 overexpression negative control; PIK3R1: PIK3R1 overexpression and miR-100-3p overexpression negative control; miR-100-3p+PIK3R1: miR-100-3p overexpression and PIK3R1 overexpression.

To more fully evaluate the functional relationship between miR-100-3p and PIK3R1 in the context of hMSC adipogenesis, we next co-transduced these cells with lentiviral vectors designed to overexpress both miR-100-3p and PIK3R1 (Figure 5A). We found that PIK3R1 overexpression was sufficient to reverse the ability of miR-100-3p overexpression to inhibit adipogenic marker gene expression (Figure 5B, 5C). These results were further supported by Oil Red O staining findings revealing that lipid droplet accumulation was enhanced in cells transduced with both of these overexpression vectors (Figure 5D, 5E).

### miR-100-3p modulates PI3K/AKT pathway signaling in the context of adipogenesis

**Figure 6:**
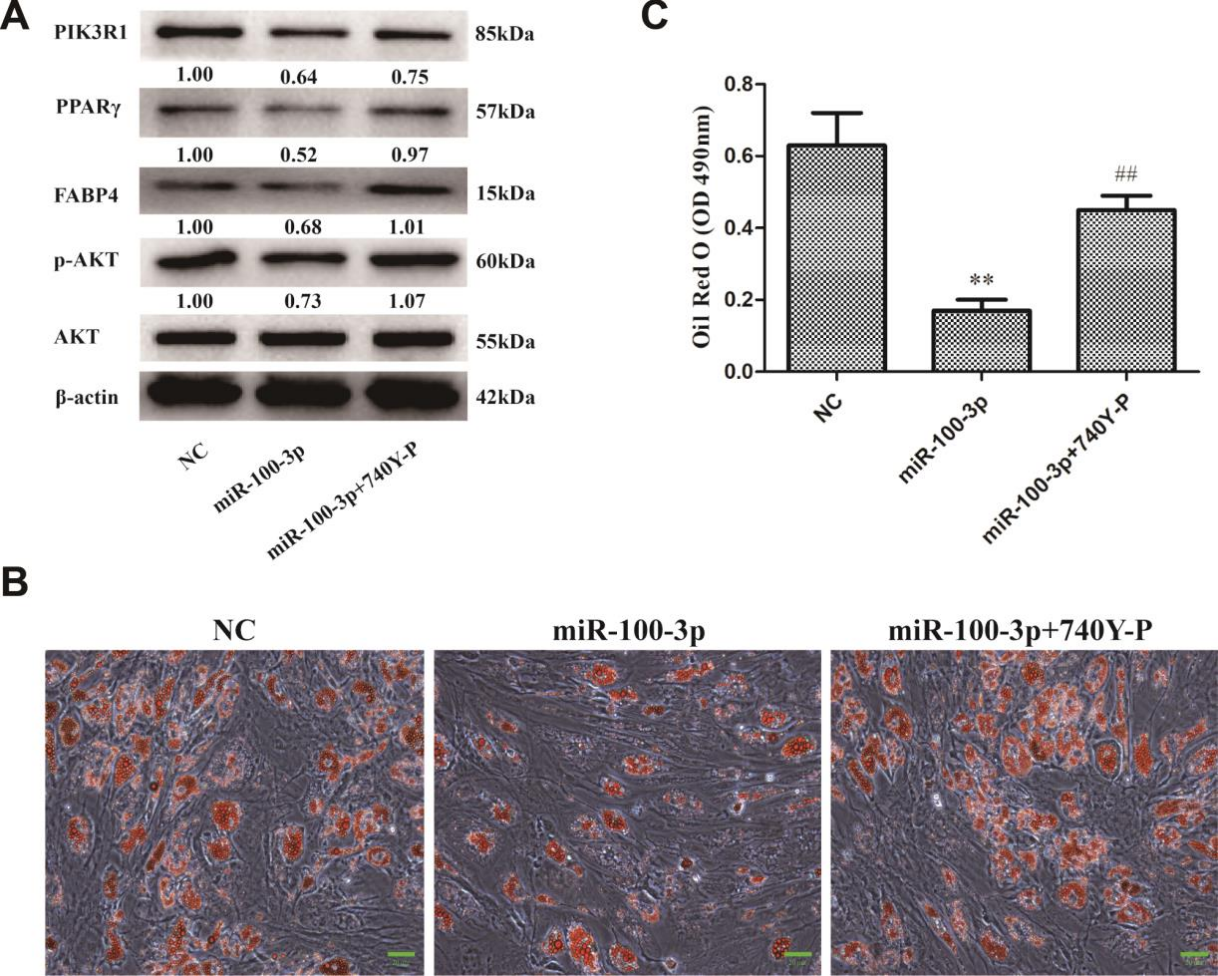
**miR-100-3p controls hMSC adipogenesis via the PI3K/AKT signaling pathway. hMSCs overexpressing miR-100-3p were treated with a PI3K/AKT signaling pathway agonist.** (**A**) Protein expression in these cells was assessed. (**B**) Lipid droplets were detected in these cells via Oil Red O staining; scale bar, 20 μm. (**C**) Oil Red O staining intensity differed significantly among study groups. Data were collected on day 7 post-adipogenic induction. Data are means ± SD (X ± SD, n=3). **P<0.01 vs negative control, ^##^ *P*<0.01 vs miR-100-3p overexpression. Note: NC: negative control; miR-100-3p: miR-100-3p overexpression, 740Y-P: PI3K/AKT agonist 740Y-P.

In order to explore the impact of miR-100-3p on PI3K/AKT pathway signaling during hMSC adipogenesis, we next assessed p-AKT levels in these cells via Western blotting, revealing that miR-100-3p overexpression was linked to decreased p-AKT levels compared to those observed in control cells (Figure 6A). To further confirm this result, a rescue experiment was performed wherein miR-100-3p-overexpressing cells were treated with the PI3K agonist 740Y-P. This co-treatment was sufficient to restore AKT phosphorylation in miR-100-3p-overexpressing cells during adipogenesis, as evidenced by increases in both adipogenic marker gene expression (Figure 6A) and Oil Red O staining (Figure 6B, 6C). Together, these findings suggest that miR-100-3p inhibits adipogenesis in hMSCs via the PIK3R1/AKT axis.

## DISCUSSION

Obesity has become an increasingly prevalent cause of morbidity and mortality globally, leading to the intensive study of the mechanistic basis for adipogenesis [18–20]. The process of adipocyte commitment and differentiation is complex, and miRNAs have been found to play important regulatory roles in this process and the context of obesity [21–22]. However, only a limited subset of miRNAs have been identified as adipogenic regulators in human cells, emphasizing the importance of more broadly studying miRNAs with regulatory roles in this important biological context.

In the present study, we found that miR-100-3p is significantly downregulated in the context of hMSC adipogenesis (Figure 1), leading us to hypothesize that it may be a key regulator of this process. We then prepared hMSCs that stably overexpressed miR-100-3p, and found that such overexpression was linked to impaired adipogenic differentiation as evidenced by reduced adipogenic marker gene expression at the mRNA and protein levels (Figure 3C, 3D). These results thus confirmed that miR-100-3p serves to negatively regulate hMSC adipogenesis.

In prior studies, miR-100-3p has been found to regulate cellular apoptosis and proliferation in gastric cancer, and it has further been leveraged as a diagnostic and therapeutic biomarker in gastric and esophageal cancer [12, 23]. However, there have not been any previous studies assessing how miR-100-3p impacts hMSC adipogenic differentiation, and ours is the first to demonstrate the central role of this miRNA in this process.

In an effort to establish the mechanisms whereby miR-100-3p controls adipogenesis as a means of highlighting novel therapeutic approaches to treating obesity, we utilized predictive bioinformatics algorithms to identify PIK3R1 a putative miR-100-3p target gene. We then confirmed this targeting relationship based upon sequence complementarity, luciferase reporter assays, and the fact that PIK3R1 was downregulated in cells overexpressing this miRNA (Figure 4C, 4D). Together our findings provided robust evidence supporting the identity of PIK3R1 as a miR-100-3p target gene.

PIK3R1 encodes the 85-kD regulatory subunit p85α of class I PI3K [24–25]. Mutations in the PIK3R1 gene have been linked to insulin resistance, cancer, and immunodeficiencies [26–28]. However, prior studies have not evaluated the functional role of PIK3R1 in the context of adipogenesis. Through rescue experiments, we confirmed that PIK3R1 overexpression was sufficient to reverse miR-100-3p-mediated suppression of hMSC adipogenesis (Figure 5B–5E), confirming that PIK3R1 plays important roles in this biological context.

**Figure 7:**
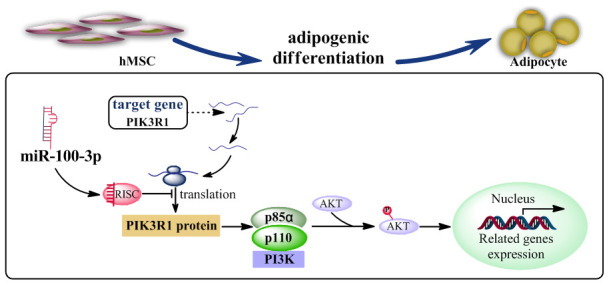
**A proposed model whereby miR-100-3p inhibits hMSC adipogenesis via targeting PIK3R via the PI3K/AKT signaling pathway.**

PI3K/AKT signaling serves as a master regulator of preadipocyte-to-adipocyte differentiation [15], with PIK3R1 being a critical component in this signaling pathway. We found that miR-100-3p overexpression reduced AKT phosphorylation in the context of adipogenesis, and we further determined that the PI3K/AKT signaling pathway agonist 740Y-P was able to enhance adipogenic differentiation in cells overexpressing miR-100-3p (Figure 6). Based upon these results, we thus confirmed that miR-100-3p is an important negative regulator of adipogenesis in hMSCs and that it functions via targeting PIK3R1 so as to modulate the PI3K/AKT signaling pathway (Figure 7).

In conclusion, we clarified the importance of miR-100-3p in hMSC adipogenic differentiation, revealing that this miRNA inhibits adipogenesis by targeting PIK3R1 via the PI3K/Akt signaling pathway. These findings may offer novel opportunities to develop therapeutic regimens for the treatment of senile obesity or other related diseases.

## MATERIALS AND METHODS

### Cell culture and adipogenesis

Flow cytometry was used to confirm that hMSCs (HUXMA-01001, Cyagen Biosciences, China) were ≥ 95% CD73-, CD90-, and CD105-positive, and were negative (≤ 5%) for CD11b, CD19, CD34, CD45, and CD HLA-DR surface expression. These cells were cultured at a density of 5 × 10^4^ cells/cm^2^ in OriCell hMSC Growth Medium (HUXMA-90011, Cyagen Biosciences) containing 10% FBS, penicillin/streptomycin, and glutamine in a humidified 5% CO_2_ incubator at 37°C. Every 3-4 days, cells were passaged with 0.25% trypsin-EDTA solution (Invitrogen, USA), and cells from passage 6 were used for all experiments in the present study.

Once confluent, hMSCs were cultured for two further days after which media was exchanged for DMEM containing 10% FBS, 10 μg/ml insulin, 0.5 mM 3-isobutyl-1-methylxanthine, and 0.5 mM dexamethasone (all from Gibco, USA) in order to initiate adipogenesis. This differentiation was allowed to proceed for 7-14 as indicated in appropriate experiments, with media being exchanged every third day. In addition, cells were collected to assess the expression of peroxisome proliferator-activated receptor γ (PPARγ), Fatty Acid Binding Protein-4 (FABP4) and PIK3R1 at the mRNA and protein levels.

### Lentiviral transduction

Lentiviral vectors used for PIK3R1 and miR-100-3p overexpression and appropriate control vectors were from Shanghai Genechem Co., Ltd. Transduction efficiency was evaluated using lentiviruses encoding green fluorescence protein (GFP), and lentiviral titers were determined via serial dilution. For transduction, hMSCs were added to 6-well plates and grown until 20-30% confluent, at which time 1 × 10^8^ TU/ml of the appropriate lentivirus (10 μl) and 5 μg/ml polybrene were added to each well in complete media. Cells were cultured for 10 h, after which media was exchanged for fresh media, and cells were cultured for 72 further hours. In order to screen for successfully transduced cells, media containing 0.5 μg/ml puromycin was utilized to culture cells after 48 h, with selection being maintained for 6 days during which time media was refreshed every 1-2 days.

### Oil red O staining and lipid quantification

At appropriate time points, cells were washed using PBS prior to fixation at room temperature for 30 minutes with 4% formalin. Cells were then washed two more times using PBS, after which they were stained for 30 minutes using 60% saturated Oil Red O. Cells were then washed two more times prior to microscopic evaluation (Olympus IX73, Tokyo, Japan). After imaging, isopropanol was used to elute dye from these cells, and absorbance at 490 nm was measured via microplate reader (Biorad iMARK™, USA) as a means of quantifying the accumulation of intracellular lipid droplets.

### qRT-PCR

Trizol (Invitrogen) was employed to extract cellular RNA based upon provided directions, after which a Reverse Transcription System and Oligo (dT) (Thermo Scientific) were utilized for cDNA preparation. The expression of miR-100-3p was quantified using a real-time PCR miRNA kit (Ribobio, China), with the U6 small RNA serving as a normalization control using appropriate primers from Ribobio. In contrast, β-actin mRNA expression served as a normalization control for mRNA expression using primers shown in Supplementary Table 1. A SYBR Premix Ex Taq kit (TOYOBO, Japan) was used for qRT-PCR reactions with a 7500 Real-Time PCR System (ABI, CA, USA), and the 2^-ΔΔCT^ approach was used for data analysis.

### Western blotting

RIPA buffer was used to lyse cells on ice, after which lysates were boiled for 5 minutes in 5×SDS sample buffer, followed by SDS-PAGE separation and transfer to PVDF membranes (Millipore). Blots were then blocked using non-fat milk prior to being probed using appropriate primary rabbit antibodies specific for PI3K (Cat. no. AF6241), AKT (Cat. no. AF6261), phospho-AKT (Cat. no. AF0016), PPARγ (Cat. no. AF6284), FABP4(AP2) (Cat. no. ab92501; Abcam), and β-actin (1:2000; Cat. no. 20536-1-AP; Proteintech). All antibodies were from Affinity and were used at a 1:1000 dilution unless otherwise noted. Anti-rabbit HRP-conjugated IgG (1:10000; Cat. no. SA00001-2; Proteintech, USA) was utilized as a secondary antibody to probe blots, after which protein bands were visualized via chemiluminescence. The normalized values under each bands were the intensity of these bands relative to β-actin or total AKT as loading controls.

### Prediction of miRNA target genes

The TargetScan 6.2 (http://www.targetscan.org/), PicTar (http://pictar.mdc-berlin.de/), and miRBase 21 (http://www.mirbase.org/) algorithms were utilized to predict potential miR-100-3p target genes.

### Luciferase reporter assay

The Dual-Luciferase Reporter Assay System (pGL3 vector; Promega, USA) was used to confirm the identity of PIK3R1 as a putative miR-100-3p target gene. Briefly, the PIK3R1 3’-UTR region containing the putative miR-100-3p binding site was cloned into the pGL3 vector downstream of the luciferase gene, with both wild-type (WT) and mutant (MUT) forms of this vector being prepared. Vectors were sequenced to verify they had been constructed appropriately, after which they were transfected into 293T cells with or without miR-100-3p mimics. The Dual-Luciferase Reporter Assay System was then used based on provided directions at 48 h post-transfection, with Renilla luciferase activity being used to normalize data and with results being calculated relative to levels on control miRNA-transfected cells.

### Statistical analysis

Data are means ± SD from triplicate experiments, and were analyzed with SPSS v16.0. Student’s t-tests were employed to compare data, with *P* < 0.05 as the significance threshold.

## Supplementary Material

Supplementary Table 1
