# Supplementary material for: miR-100-3p inhibits the adipogenic differentiation of hMSCs by targeting PIK3R1 via the PI3K/AKT signaling pathway
Source: Aging (Albany NY). 2020 Nov 20;12(24):25090–100. doi: 10.18632/aging.104074 (PMC7803504; doi:10.18632/aging.104074)
Supplement: Supplementary Table 1 [file aging-12-104074-s001.pdf]

SUPPLEMENTARY TABLES

Supplementary Table 1. Primers used in this study forβ-actin, PPARγ, FABP4, and PIK3R1.

Gene symbol	Forward primer	Reverse primer
PPARγ	5'- GGGATGTCTCATAATGCCATCAG- 3'	5'-GCCCTCGCCTTTGCTTTG-3'
FABP4	5-'GGATGATAAACTGGTGGTGGAATG-3'	5'- CAGAATGTTGTAGAGTTCAATGCGA -3'
PIK3R1	5-'GAAGCGAGATGGCACTTTTC-3	5-' AGCCATAGCCAGTTGCTGTT-3
β-actin	5'-GCGAGAAGATGACCCAGATCATGT-3'	5'-TACCCCTCGTAGATGGGCACA-3'
